# Quantitative *T*_1_ brain mapping in early relapsing-remitting multiple sclerosis: longitudinal changes, lesion heterogeneity and disability

**DOI:** 10.1007/s00330-023-10351-6

**Published:** 2023-11-09

**Authors:** James G. Harper, Elizabeth N. York, Rozanna Meijboom, Agniete Kampaite, Michael J. Thrippleton, Patrick K. A. Kearns, Maria del C. Valdés Hernández, Siddharthan Chandran, Adam D. Waldman, On behalf of the FutureMS consortium, Amit Akula, Sergio Baranzini, Fiona Barret, Mark Bastin, Chris Batchelor, Emily Beswick, Fraser Brown, Tracy Brunton, Javier Carod Artal, Jessie Chang, Yingdi Chen, Shuna Colville, Peter Connick, Annette Cooper, Denise Cranley, Rachel Dakin, Baljean Dhillon, Liz Elliott, James Finlayson, Peter Foley, Stella Glasmacher, Angus Grossart, Haane Haagenrud, Katarzyna Hafezi, Emily Harrison, Adil Harroud, Sara Hathorn, Tracey Hopkins, David Hunt, Aidan Hutchison, Charlotte Jardine, Kiran Jayprakash, Matt Justin, Gwen Kennedy, Lucy Kessler, Michaela Kleynhans, Juan Larraz, Katherine Love, Dawn Lyle, James MacDonald, Niall MacDougall, Jen MacFarlane, Lesley Macfarlane, Alan Maclean, Bev MacLennan, Margaret-Ann MacLeod, Nicola Macleod, Don Mahad, Sarah-Jane Martin, Conni McCarthy, Lynn McMahon, Daisy Mollison, Ian Megson, Daisy Mollison, Mary Monaghan, Lee Murphy, Katy Murray, Judith Newton, Julian Ng Kee Kwong, Jonathan O’Riordan, David Perry, Suzanne Quigley, Adam Scotson, Scott Semple, Amy Stenson, Michaela Stuart, Christine Weaver, Stuart Webb, Belinda Weller, Nicole White, Anna Williams, Stewart Wiseman, Charis Wong, Michael Wong, Rosie Woodward

**Affiliations:** 1 Centre for Clinical Brain Sciences, University of Edinburgh, Edinburgh BioQuarter: Chancellors Building, Edinburgh, EH16 4SB UK; 2 Edinburgh Imaging, University of Edinburgh, Edinburgh, UK; 3 Anne Rowling Regenerative Neurology Clinic, Edinburgh, UK; 4 UK Dementia Research Institute, University of Edinburgh, Edinburgh, UK

**Keywords:** Magnetic resonance imaging, Multiple sclerosis (Relapsing-Remitting), Longitudinal studies, Brain, Multiparametric magnetic resonance imaging

## Abstract

**Objectives:**

To quantify brain microstructural changes in recently diagnosed relapsing-remitting multiple sclerosis (RRMS) using longitudinal *T*_1_ measures, and determine their associations with clinical disability.

**Methods:**

Seventy-nine people with recently diagnosed (< 6 months) RRMS were recruited from a single-centre cohort sub-study, and underwent baseline and 1-year brain MRI, including variable flip angle *T*_1_ mapping. Median *T*_1_ was measured in white matter lesions (WML), normal-appearing white matter (NAWM), cortical/deep grey matter (GM), thalami, basal ganglia and medial temporal regions. Prolonged *T*_1_ (≥ 2.00 s) and supramedian *T*_1_ (relative to cohort WML values) WML voxel counts were also measured. Longitudinal change was assessed with paired *t*-tests and compared with Bland-Altman limits of agreement from healthy control test-retest data. Regression analyses determined relationships with Expanded Disability Status Scale (EDSS) score and dichotomised EDSS outcomes (worsening or stable/improving).

**Results:**

Sixty-two people with RRMS (mean age 37.2 ± 10.9 [standard deviation], 48 female) and 11 healthy controls (age 44 ± 11, 7 female) contributed data. Prolonged and supramedian *T*_1_ WML components increased longitudinally (176 and 463 voxels, respectively; *p* < .001), and were associated with EDSS score at baseline (*p* < .05) and follow-up (supramedian: *p* < .01; prolonged: *p* < .05). No cohort-wide median *T*_1_ changes were found; however, increasing *T*_1_ in WML, NAWM, cortical/deep GM, basal ganglia and thalami was positively associated with EDSS worsening (*p* < .05).

**Conclusion:**

*T*_1_ is sensitive to brain microstructure changes in early RRMS. Prolonged WML *T*_1_ components and subtle changes in NAWM and GM structures are associated with disability.

**Clinical relevance statement:**

MRI *T*_1_ brain mapping quantifies disability-associated white matter lesion heterogeneity and subtle microstructural damage in normal-appearing brain parenchyma in recently diagnosed RRMS, and shows promise for early objective disease characterisation and stratification.

**Key Points:**

*• Quantitative T*_*1*_ *mapping detects brain microstructural damage and lesion heterogeneity in recently diagnosed relapsing-remitting multiple sclerosis.*

*• T*_*1*_ *increases in lesions and normal-appearing parenchyma, indicating microstructural damage, are associated with worsening disability.*

*• Brain T*_*1*_ *measures are objective markers of disability-relevant pathology in early multiple sclerosis.*

**Graphical abstract:**

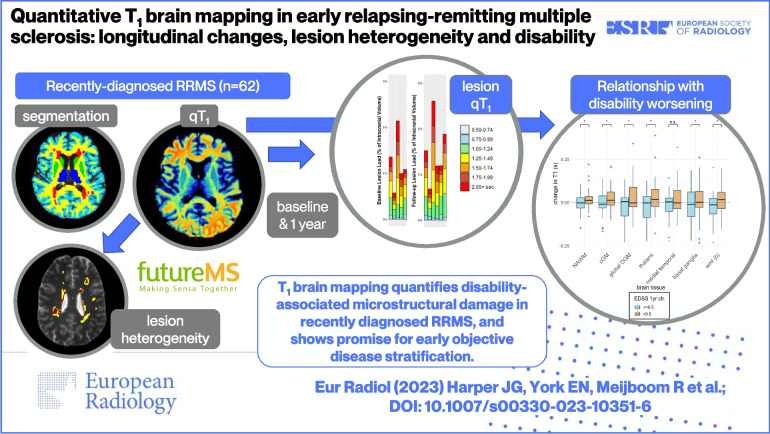

**Supplementary Information:**

The online version contains supplementary material available at 10.1007/s00330-023-10351-6.

## Introduction

Disease trajectory in relapsing-remitting multiple sclerosis (RRMS) is highly variable, and difficult to predict early in the disease course. Improved objective prognostic markers, which are sensitive to early RRMS pathological features, are needed to better inform early treatment decisions from the point of diagnosis, and for future clinical trials.

Episodic immune-mediated demyelination is thought to result in axonal damage and neuronal dysfunction [1]. Identification of new or enlarging white matter lesions (WML) visible on *T*_2_-weighted or *T*_2_-FLAIR (fluid-attenuated inversion recovery) MRI is widely used as an indicator of interval inflammatory disease activity [2]. The number and volume of WML do not, however, adequately explain clinical disability in RRMS [3, 4]. Microscopic damages to grey matter (GM) and normal-appearing white matter (NAWM) are also pathological features of RRMS, [5] and heterogeneity of microstructural damage within WML that appears similar on *T*_2_-weighted MRI is observable using quantitative microstructure-sensitive MRI methods [6–9]*.*

A subset of *T*_2_-weighted WML are visibly hypointense on *T*_1_-weighted MRI, reflecting *T*_1_ prolongation, [10] and have variously been described as ‘black hole’ or ‘*T*_1_w-hypointense’ lesions [11]. Such ‘*T*_1_w-hypointense’ lesions are thought to represent more severely damaged white matter [12] and are associated with worse clinical disability [13, 14]. Radiological evaluation of *T*_1_-weighted hypointensity is nevertheless subjective and qualitative, and will not capture intralesional *T*_1_ heterogeneity or subtle change in normal-appearing tissue.

The spin-lattice relaxation time (*T*_1_) of protons in brain parenchyma is inversely proportional to myelin density [11]. Previous imaging-histological correlation indicates that *T*_1_ quantification may provide a non-invasive marker of myelin density in multiple sclerosis (MS), [12] and is sensitive to subtle abnormalities in normal-appearing GM and NAWM in RRMS [5, 15–20]. Widespread *T*_1_ prolongation is seen in RRMS compared with healthy controls, and correlates with disability [14, 20, 21]. Quantitative mapping of *T*_1_ may also distinguish microstructural heterogeneity within WML; substantially prolonged *T*_1_ tissue volumes appear to be a better predictor of disability than visual assessment of *T*_1_-weighted hypointense WML [22]. The contribution of prolonged *T*_1_ components in WML and other brain tissues to clinical disability in early disease, and how these evolve with time remain to be explored.

The primary aims of this study were to assess whether changes in tissue microstructure integrity, reflected in *T*_1_ heterogeneity and prolongation, are quantifiable within WML and other brain regions over the first year following RRMS diagnosis, and to ascertain the relationship between *T*_1_ prolongation and clinical disability. We hypothesised that prolonged *T*_1_ components would progress with time and be associated with worsening clinical disability.

Our secondary exploratory aims were to examine intralesional *T*_1_ heterogeneity and its relationship with disability, investigate single-time point measures as predictors of clinical disability trajectory following RRMS diagnosis and establish the test-retest reliability of *T*_1_ measures.

## Materials and methods

Ethics approval was obtained from the South East Scotland Research Ethics Committee 02 (REC 15/SS/0233). The study conformed to the Declaration of Helsinki 2000 (amendments in 2002 and 2004) and Good Clinical Practice ICH guidelines. All participants gave written informed consent and data were pseudo-anonymised.

### Participants

**Fig. 1:**
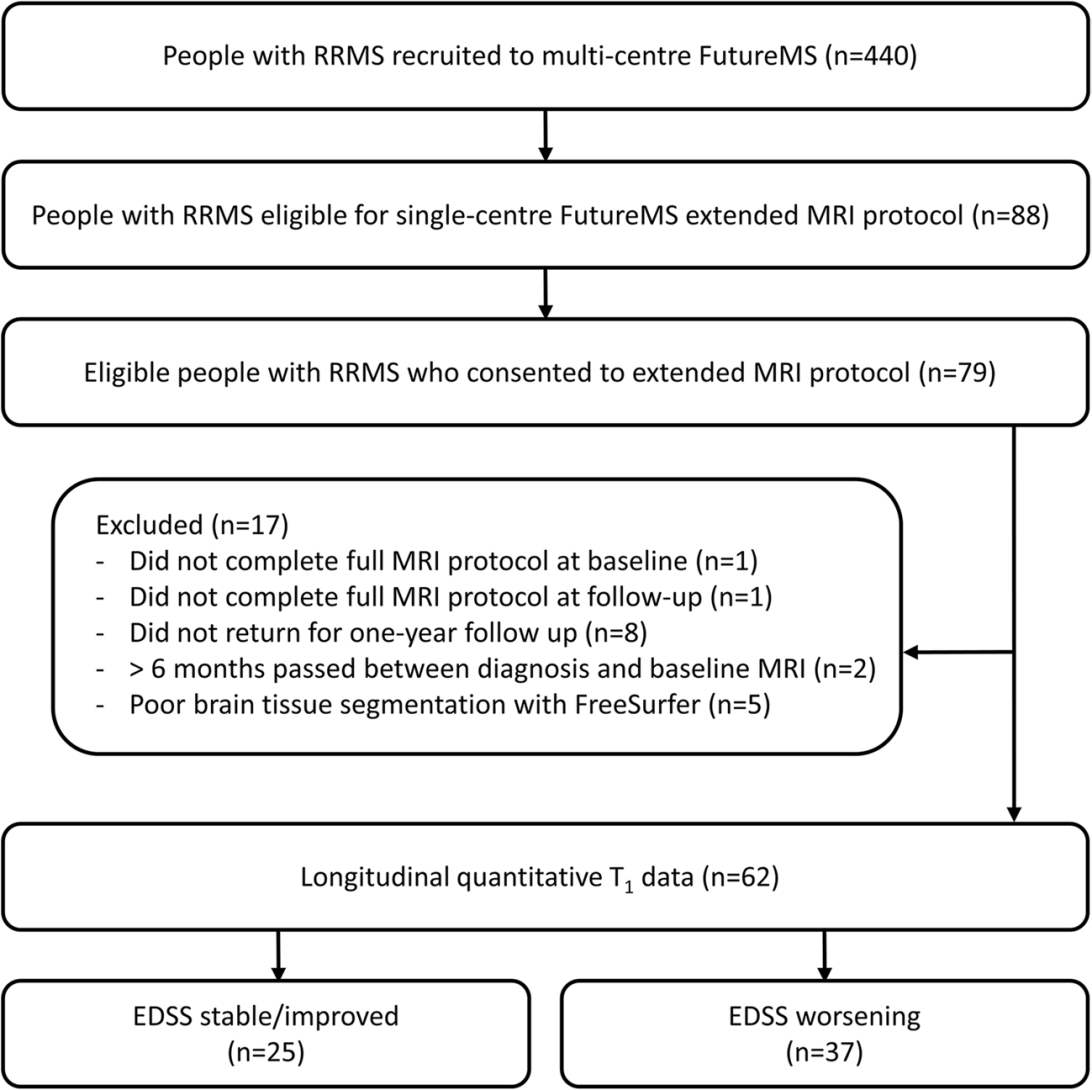
Flowchart showing inclusion and exclusion criteria for FutureMS quantitative *T*_1_ sub-study

Seventy-nine patients were recruited to a single-centre sub-study of FutureMS, a prospective multi-centre longitudinal inception cohort study of people with recently diagnosed RRMS across Scotland (Fig. 1) [23, 24]. Inclusion criteria were as follows: < 6 months from diagnosis; aged ≥ 18 years; and capacity to provide informed consent. Exclusion criteria were as follows: previous use of disease-modifying therapies (DMTs); clinical trial participation prior to baseline assessment; and contraindications for MRI. FutureMS sample size calculation has been previously described; [24] Edinburgh participants were pragmatically offered extended MRI once the sub-study recruitment opened in November 2017 until March 2019. Twelve age- and sex-matched healthy controls were recruited for test-retest purposes. Exclusion criteria were contraindication for MRI and incidental MRI findings.

MRI and Expanded Disability Status Scale (EDSS) score were acquired at baseline (between November 2017 and July 2019) and 1-year follow-up. Handling of missing data and loss to follow-up are detailed elsewhere; briefly, data missing at random were imputed using predictive mean matching [23].

### MRI protocol

MRI was performed on a 3-Tesla Siemens MAGNETOM Prisma clinical system at the University of Edinburgh Imaging Facility. Whole-brain *T*_1_ mapping was performed using a variable flip angle (VFA) 3D multi-echo spoiled gradient recalled echo sequence (total acquisition time: 12 min 28 s) as part of a comprehensive MRI protocol, including a 2D *T*_2_-weighted FLAIR and 3D *T*_1_-weighted MPRAGE (Table S1) [24]. Control subjects underwent an identical MRI protocol, repeated within 2 weeks, to determine test-retest agreement.

### Structural MRI data processing

**Fig. 2:**
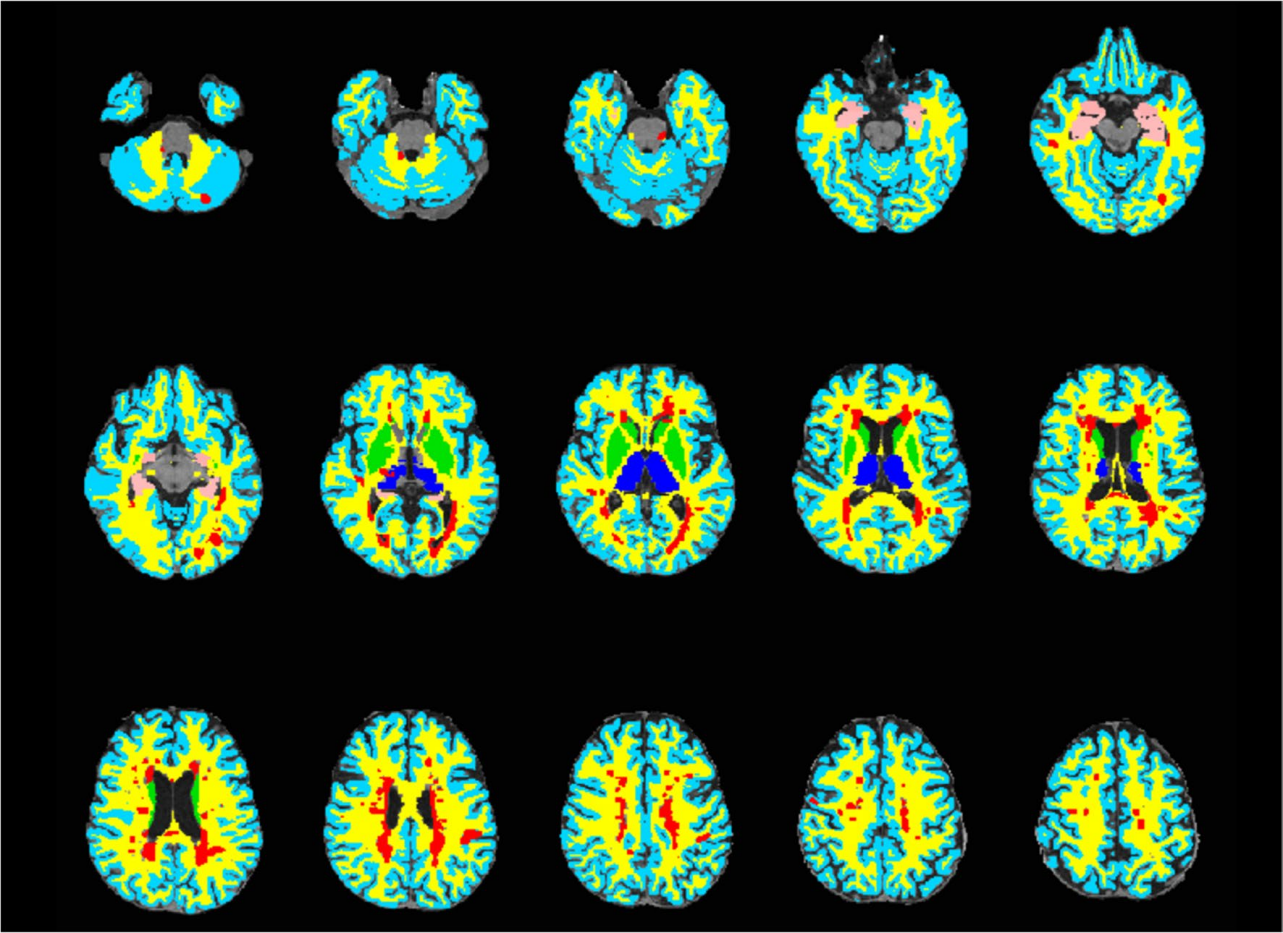
An axial *T*_1_ map with superimposed brain tissue segmentations: normal-appearing white matter (yellow), white matter lesions (red), cortical grey matter (light blue), thalami (dark blue), basal ganglia (green) and medial temporal regions (pink)

WML segmentation was performed on baseline 2D *T*_2_ FLAIR images using an adaptation of a previously reported thresholding method, [25] with manual correction [24]. Tissues were segmented with FreeSurfer (v6.0, http://surfer.nmr.mgh.harvard.edu/) from the *T*_1_-weighted MPRAGE [24]. These included NAWM; cortical GM (cGM); medial temporal regions (hippocampi and amygdala); basal ganglia (caudate, pallidum and putamen); thalami; and global deep GM (DGM; combined thalami, basal ganglia and medial temporal regions; Fig. 2). WML load was calculated as the percentage of occupied intracranial volume [24].

### *T*_1_ data processing

**Fig. 3:**
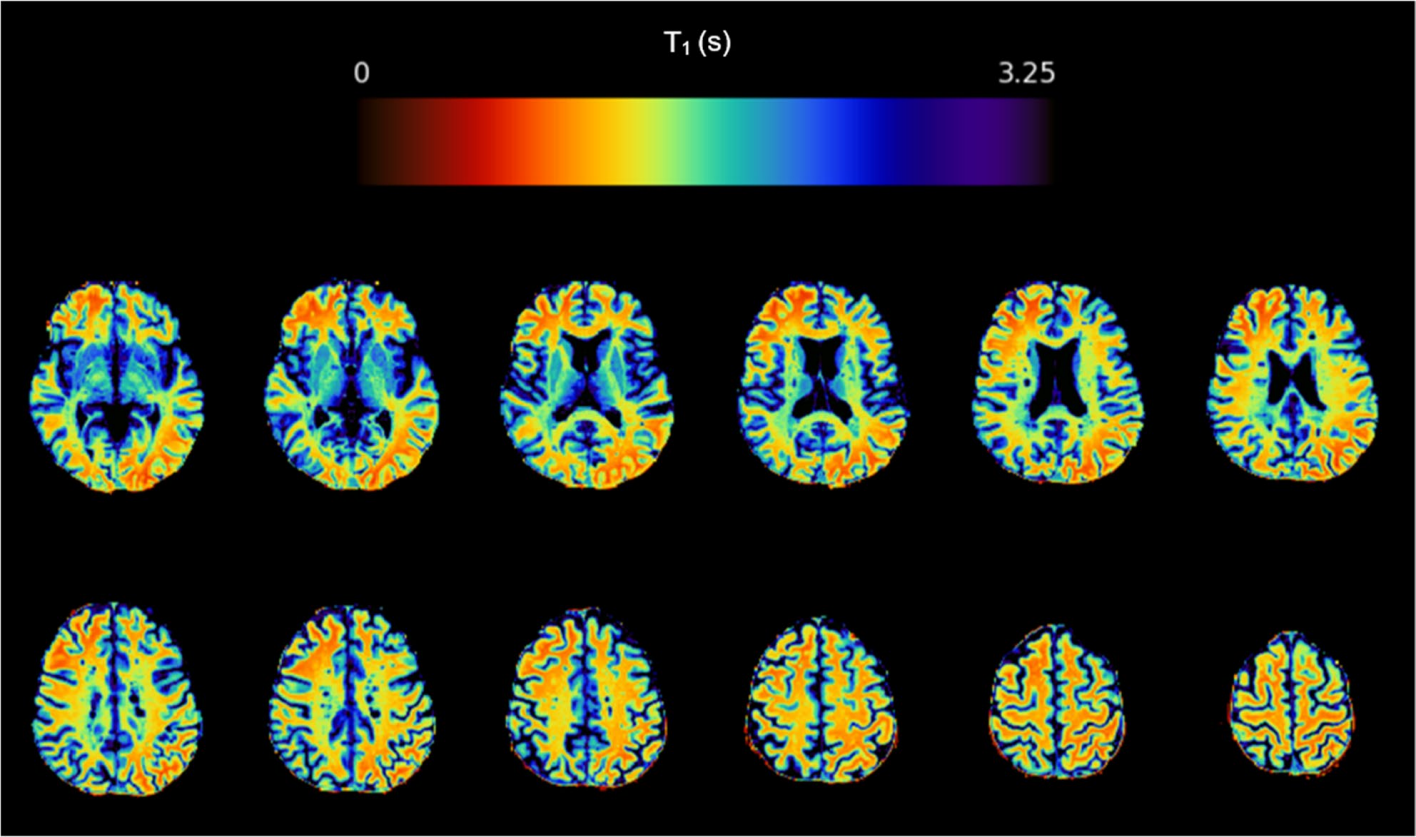
Axial *T*_1_ (colour) map brain slices for a person with recently diagnosed relapsing-remitting multiple sclerosis taking part in FutureMS. The colour bar represents *T*_1_ in seconds of the colour scale

*T*_1_ parametric maps were obtained from VFA gradient echo images in MATLAB (R2018b) using previously described equations (Fig. 3; software available: https://doi.org/10.7488/ds/2965) [26, 27]. Tissue segmentations were registered to *T*_1_ maps using FSL (v6.0.1) FLIRT [28].

Median *T*_1_ within each tissue was calculated per patient (fslstats). To investigate *T*_1_ WML heterogeneity, the proportion of WML voxels within each 0.25-s interval between 0.5 and 2.00 s was calculated for each patient at baseline and follow-up (RStudio v4.0.2).

As *T*_1_ prolongation is associated with demyelination [12] and disability, [3] we also characterised WML components with relatively prolonged *T*_1_ using a thresholding approach [22]. The number of WML voxels with (1) *T*_1_ ≥ 2.00 s (here termed ‘prolonged *T*_1_’) and (2) supramedian *T*_1_ (calculated per patient relative to the baseline cohort mean of WML median *T*_1_) was thus calculated at each time point.

### Statistical analysis

All statistical analyses were performed in RStudio (v4.0.2).

#### Test-retest healthy controls

To establish test-retest reference levels, Bland-Altman limits of agreement were calculated from healthy control data, following previously reported methodology [29]. To assess whether the difference between time points was statistically significant, sign tests were performed.

#### Longitudinal change in *T*_1_ metrics

Paired two-sided *t*-tests were used to examine longitudinal changes in median *T*_1_ for each tissue and, as separate analyses, 1-year changes in the number of prolonged/supramedian *T*_1_ voxels and WML load. To account for WML voxels forming during the 1-year follow-up period, which could positively skew WML *T*_1_ data, a post hoc paired *t*-test was performed including only WML voxels already present at baseline.* T*_1_ changes in NAWM and cGM were plotted against test-retest limits for visual comparison. To control for type II error, False Discovery Rate (FDR) correction (*q* < 0.05) was applied for primary analyses [30].

#### Relationship with clinical disability

The relationship between *T*_1_ and EDSS score at baseline was investigated using ordinal logistic regression. In view of limited EDSS changes within our recently diagnosed cohort, 1-year change in EDSS score was dichotomised as either worsening EDSS, defined as ≥ 0.5 points, or stable/improving; the relationship between disability groups and both baseline and 1-year changes in *T*_1_ measures was investigated using binomial logistic regression, adjusted for covariates and interactions as appropriate (Table S2 for extended methods). FDR correction was applied separately for the three sets of analyses, which are assumed to be distinct families of hypotheses [30].

Regression analyses were repeated for prolonged and supramedian *T*_1_ WML measures. The relationship between WML prolonged *T*_1_ measures at 1-year follow-up and EDSS score at follow-up was also investigated using ordinal logistic regression.

## Results

### Demographics

Complete data were available from sixty-two RRMS patients (Fig. 1), and demographics (Table 1) were comparable to the wider FutureMS cohort [23]. Six participants (*n* = 3 worsening versus *n* = 3 stable/improving disability) reported a relapse in the 6 weeks prior to baseline MRI (range: 11–34 days), one of whom received oral steroid treatment (27 days before MRI). No relapses or steroid treatments were reported in the 6 weeks prior to follow-up. At follow-up, 61% (*n* = 38) of participants had begun treatment with DMTs, mainly dimethyl fumarate (*n* = 23; Table S3). There was no significant difference in DMT status between disability groups (*Χ*^2^ = 0.79, *p* = 0.37).

**Table 1:** Cohort demographics of the relapsing-remitting multiple sclerosis (RRMS) participants. Differences between disability groups were assessed with Welch’s *t*-test for age, baseline/follow-up Expanded Disability Status Scale (EDSS) score, white matter lesion load and interval between baseline and follow-up MRI, and Pearson’s chi-squared (*Χ*^2^) test of independence with Yates’ continuity correction for sex and disease-modifying therapy (DMT) status

	Cohort	Stable/improving EDSS	Worsening EDSS
Number of RRMS participants	62	25	37
Mean age (SD) [range] in years	37.2 (10.9) [21 to 67]	36.7 (9.7) [22 to 59]	37.5 (11.9) [21 to 67]
Sex (female:male)	48:14	19:6	29:8
Median baseline EDSS score [range]	2 [0 to 6]	2 [0 to 3.5]	2 [0 to 6]
Median follow-up EDSS score [range]	2.5 [0 to 6.5]	2 *** [0 to 3.5]	3 *** [1.5 to 6.5]
Number of participants on disease-modifying therapies at 1-year follow-up	38 (61%)	17 (68%)	21 (57%)
Mean interval (SD) [range] between baseline and 1-year MRI in days	385 (35.2) [339 to 520]	383 (28.9) [339 to 451]	386 (39.2) [352 to 520]
Mean baseline lesion load (%ICV)	0.802	0.570	0.959
Mean change in lesion load	0.235	0.196	0.261

Asterisks mark significant differences: * < 0.05, *** < 0.001

*RRMS*, relapsing-remitting multiple sclerosis; *EDSS*, Expanded Disability Status Scale; *SD*, standard deviation; *ICV*, intracranial volume

One control participant was excluded due to an unexpected incidental finding; test–retest data was therefore available from eleven controls (age 44 ± 11, 7 female).

### Test-retest reliability

Test-retest agreement determined from control data is shown in Fig. S1. The difference in median *T*_1_ was not significant for all brain regions (Table S4).

### Characterising *T*_1_ in white matter lesions

**Fig. 4:**
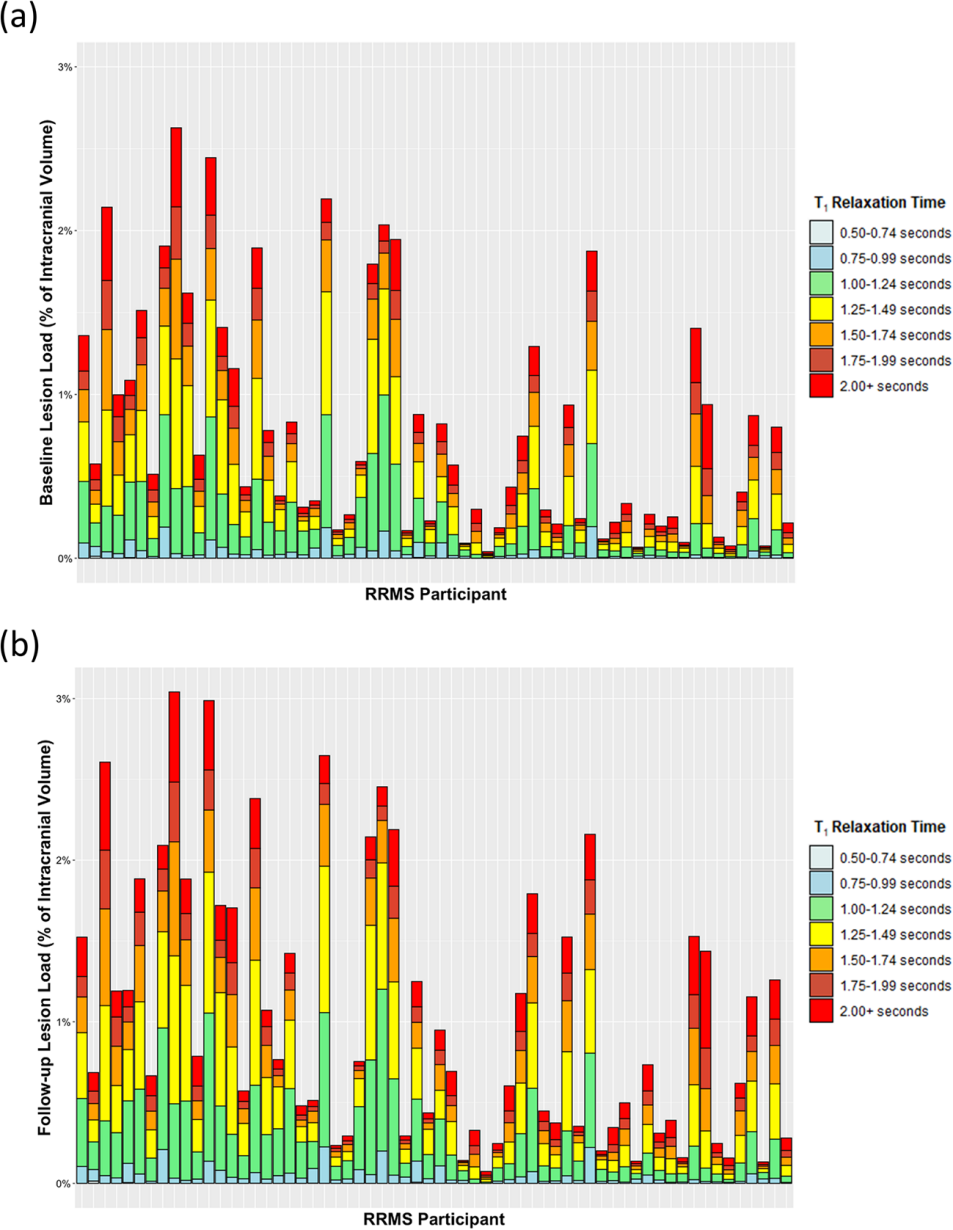
White matter lesion *T*_1_ heterogeneity in our cohort of people with recently diagnosed relapsing-remitting multiple sclerosis (RRMS) (*n* = 62). Each bar represents one participant with RRMS. The colours of the stacked plot relate to the proportion of a given participant’s white matter lesion voxels within a given *T*_1_ range at (**a**) baseline and (**b**) 1-year follow-up

**Fig. 5:**
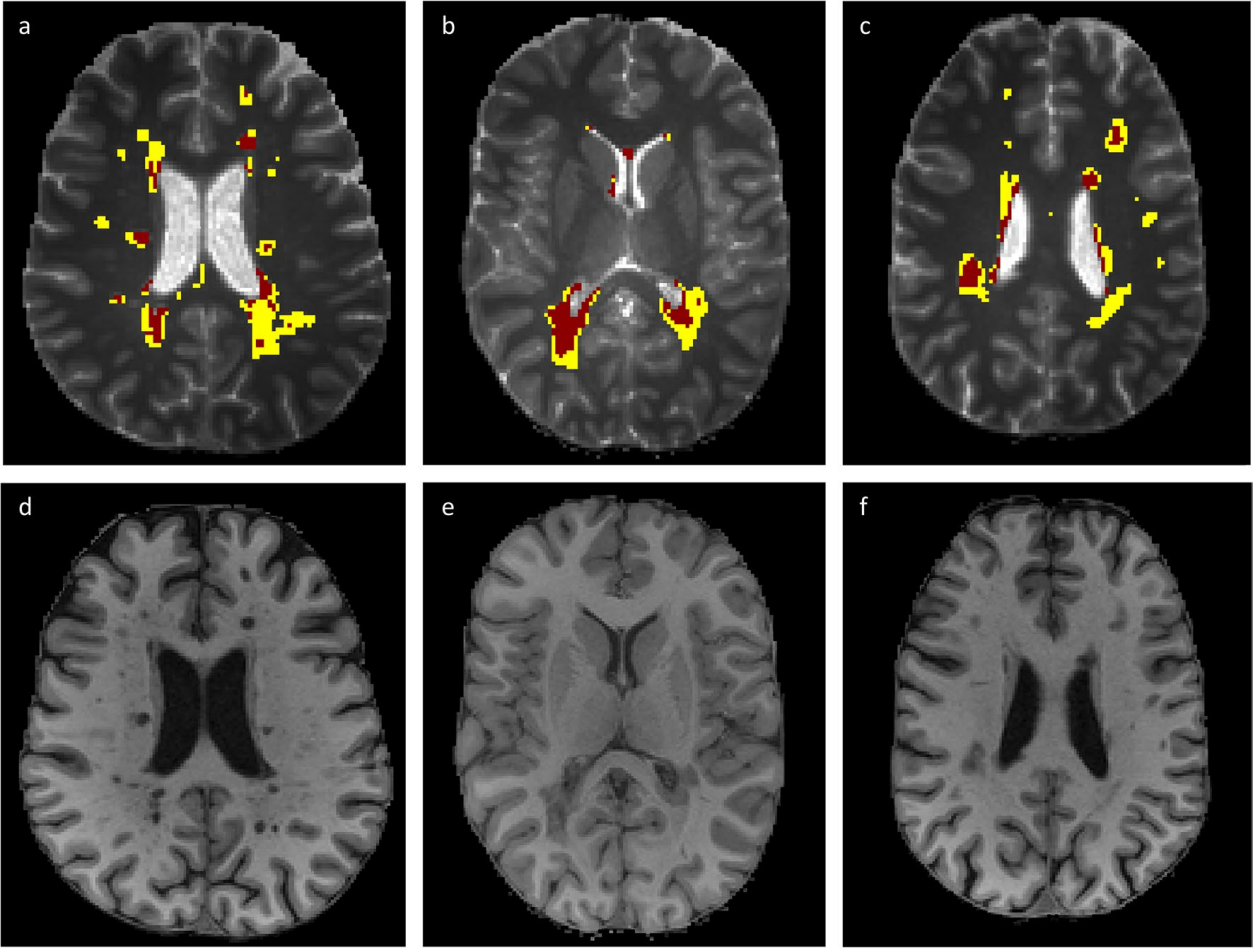
Spatial distribution (**a**–**c**) of prolonged *T*_1_ (i.e. ≥ 2.00 s) white matter lesion (WML) voxels (red) superimposed on all WML voxels (yellow) and whole-brain *T*_1_ maps (greyscale) in three representative relapsing-remitting multiple sclerosis study participants, with corresponding *T*_1_-weighted MPRAGE structural images (**d**–**f**)

Qualitative assessment showed varied proportions of WML voxels within each 0.25-s *T*_1_ range between patients, with a greater proportion in higher *T*_1_ ranges at follow-up (Fig. 4b) relative to baseline (Fig. 4a). Visual inspection showed discrete regions of relative *T*_1_ prolongation were present across WML (Fig. 5a–c), generally coinciding with *T*_1_-weighted ‘black holes’ (Fig. 5d–f).

### Longitudinal change in *T*_1_ metrics

WML median *T*_1_ decreased by a mean average of 31 ms longitudinally (*t*(61) = 2.98, FDR-corrected *p* < 0.05, 95% CI [− 52, − 10]); no other tissues showed significant longitudinal *T*_1_ changes (Table 2). Longitudinal change in NAWM and WML *T*_1_ for the majority of patient data points fell within test-retest Bland-Altman limits established in healthy controls (Fig. S2).

**Table 2:** Brain tissue median *T*_1_ summary statistics and results for paired *t*-tests

Brain tissue	*T*_1_ (ms), mean ± SD		Mean difference (ms) [95% CI]	*t*-value	*p*-value (uncorr.)	*p*-value (FDR-adj.)
	Baseline	Follow-up				
White matter lesions^a^	1462 ± 169	1431 ± 147	− 31 [− 52, − 10]	2.98	0.004	0.032*
White matter lesions^b^	1462 ± 169	1457 ± 166	− 5 [− 25, 15]	0.50	0.618	0.773
Normal-appearing white matter	1074 ± 62	1086 ± 62	11 [− 3, 26]	1.54	0.130	0.520
Cortical grey matter	1613 ± 77	1624 ± 68	10 [− 8, 29]	1.14	0.261	0.522
Global deep grey matter	1798 ± 93	1805 ± 88	7 [− 16, 31]	0.63	0.533	0.773
Basal ganglia	1679 ± 94	1682 ± 90	3 [− 19, 26]	0.29	0.773	0.773
Medial temporal region	2217 ± 109	2222 ± 96	4 [− 22, 30]	0.33	0.741	0.773
Thalami	1783 ± 90	1798 ± 88	15 [− 9, 40]	1.23	0.225	0.522

^a^Includes any voxels reclassified as lesional at 1-year follow-up

^b^Only includes lesions present at baseline

Asterisk (*) indicates a significant change in *T*_1_ over 1 year after False Detection Rate (FDR) correction

*CI*, confidence interval; *SD*, standard deviation; *uncorr.*, uncorrected for multiple comparisons

A longitudinal increase in WML load (mean average 0.235%, *t*(61) = 11.3, *p* < 0.001, 95% CI [0.193, 0.277]) prompted a post hoc analysis investigating only WML voxels present at baseline. This showed no significant change in median WML *T*_1_ (*t*(61) = 0.50, *p* = 0.618; Table 2).

There was a groupwise mean increase of 176 prolonged *T*_1_ WML voxels (*t*(61) = 5.11, *p* < 0.001, 95% CI [107, 246]) and an increase of 463 supramedian WML voxels (*t*(61) = 6.22, *p* < 0.001, 95% CI [314, 612]). When WML voxels which had formed during the follow-up period were excluded, changes in both measures remained significant (*p* < 0.001).

### Relationship with clinical disability

**Fig. 6:**
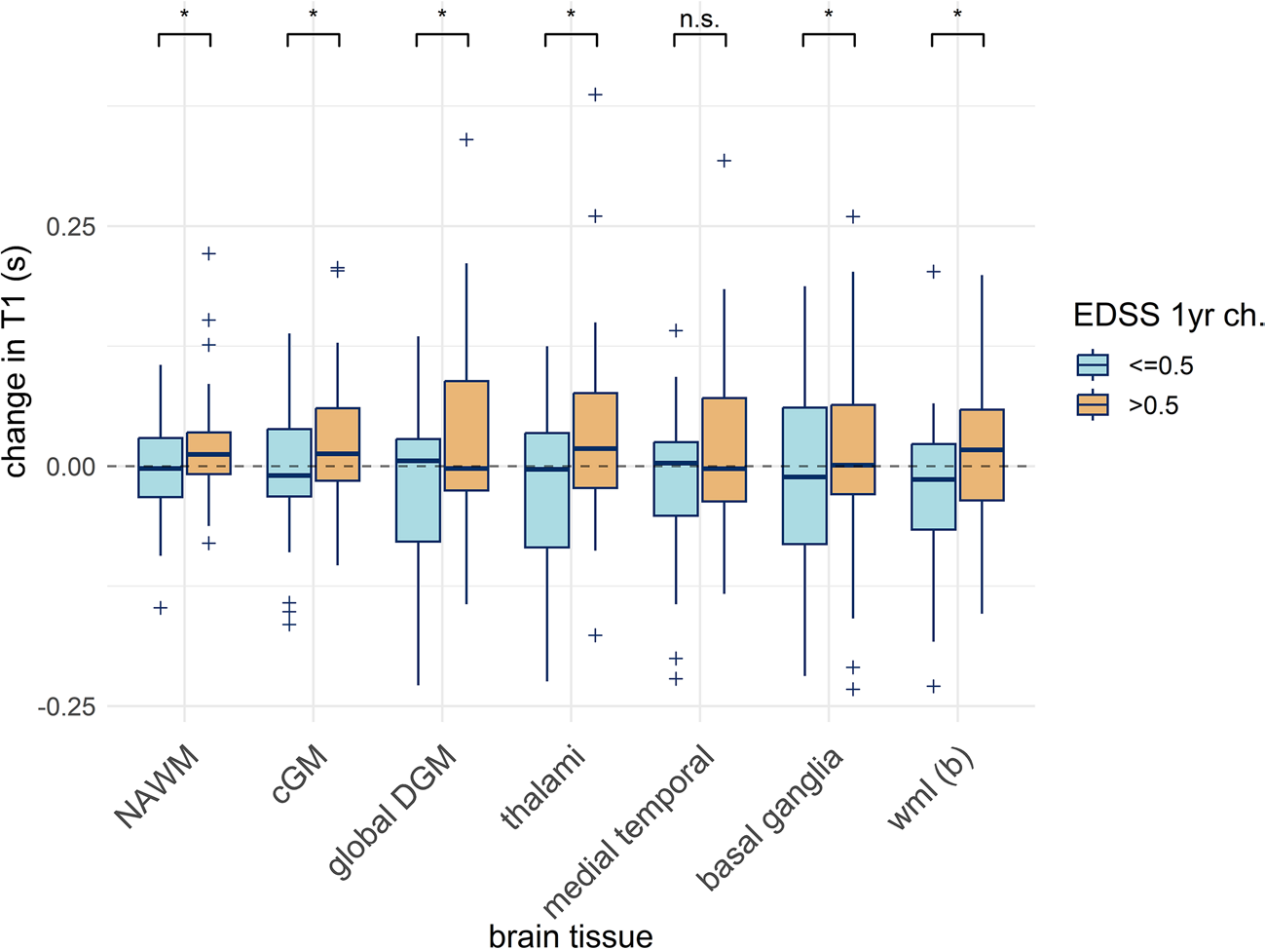
One-year change in *T*_1_ in our recently diagnosed relapsing-remitting multiple sclerosis cohort (*n* = 62). Boxplots are shown for normal-appearing white matter (NAWM), cortical grey matter (cGM), global deep grey matter (DGM), thalami, medial temporal regions, basal ganglia and white matter lesions (WML, b) including only WML voxel present at baseline. Boxplots are grouped according to dichotomised change in Expanded Disability Status Scale (EDSS) score over 1 year of  < / ≥ 0.5 points. Asterisk (*) denotes significant (*p* < .05 with False Detection Rate correction) association between difference over 1 year in *T*_1_ and disability group after adjusting for age, baseline *T*_1_, 1-year change in lesion load, disease-modifying therapy status and any significant interaction effects (see text for details); n.s., not significant

Baseline median *T*_1_ was not associated with baseline EDSS score (all* p* > 0.05; Table S5) nor with EDSS worsening (all *p* > 0.05; Table S6) in any tissue studied. One-year changes in *T*_1_ (Table S7) within NAWM, WML, cGM, DGM, the basal ganglia and thalami (but not the medial temporal regions) were however positively associated with a greater risk of EDSS worsening (all *p* < 0.05; Fig. 6, and Table 3). The relationship between change in WML *T*_1_ and EDSS worsening was mediated by an interaction with age, whereby older age strengthened the association.

**Table 3:** Relationship between 1-year change in *T*_1_ and change in disability in newly diagnosed relapsing-remitting multiple sclerosis (*n* = 62). Results are shown for binomial logistic regression models investigating the relationship between the change in median *T*_1_ in each tissue and dichotomised change in Expanded Disability Status Scale (EDSS) score (stable/improving vs worsening EDSS [< / ≥ 0.5 points]) over 1 year. Covariates were age, median baseline *T*_1_, 1-year change in white matter lesion load and disease-modifying therapy status. Continuous variables were centred and scaled, and adjusted odds ratios are for standardised data

Brain tissue	*β*	Adjusted odds ratio [95% CI]	Std. error	*Z*-value	*p*-value (uncorr.)	*p*-value (FDR-adj.)
White matter lesions^a,c^	1.284	3.61 [1.53, 11.00]	0.495	2.59	0.009	0.029*
White matter lesions^b,c^	0.998	2.71 [1.34, 6.44]	0.394	2.53	0.011	0.029*
Normal-appearing white matter	1.088	2.97 [1.35, 7.90]	0.448	2.43	0.015	0.030*
Cortical grey matter	1.236	3.44 [1.46, 9.94]	0.485	2.55	0.011	0.029*
Global deep grey matter	0.875	2.40 [1.14, 6.00]	0.417	2.10	0.036	0.041*
Basal ganglia	0.924	2.52 [1.21, 6.29]	0.416	2.22	0.026	0.035*
Medial temporal region	0.533	1.70 [0.85, 3.76]	0.372	1.44	0.151	0.151
Thalami	0.943	2.57 [1.22, 6.36]	0.417	2.26	0.024	0.035*

Asterisk (*) indicates a significant relationship at the *p* < 0.05 level after False Detection Rate (FDR) correction

^a^Includes all voxels classed as white matter lesions (WML) at follow-up

^b^Includes only voxels previously classed as WML at baseline

^c^Mediated by a significant interaction between change in WML *T*_1_ and age

*β*, standardised beta coefficient (log odds); *CI*, confidence interval; *std.*, standard; *uncorr.*, uncorrected

At baseline, greater numbers of prolonged *T*_1_ and supramedian WML voxels were independently associated with higher baseline EDSS (both *p* < 0.05; Table 4); neither measure was predictive of EDSS worsening (Table 5). One-year changes in prolonged *T*_1_ and supramedian WML voxel counts were positively associated with EDSS worsening (*p* < 0.05 and *p* < 0.01 respectively; Table 5). Prolonged and supramedian WML *T*_1_ measures at follow-up were also positively related with follow-up EDSS score (*p* < 0.05 and *p* < 0.01 respectively; Table 4).

**Table 4:** Cross-sectional relationship between prolonged/supramedian *T*_1_ white matter lesion (WML) voxel count and disability in newly diagnosed relapsing-remitting multiple sclerosis (*n* = 62). Results are shown for ordinal logistic regression models investigating the relationship at (a) baseline and (b) 1-year follow-up between the number of prolonged/supramedian *T*_1_ WML voxels and Expanded Disability Status Score (EDSS). WML voxels were classed (non-exclusively) as prolonged *T*_1_, where *T*_1_ ≥ 2.00 s, and supramedian *T*_1_, where *T*_1_ ≥ cohort mean of baseline median WML *T*_1_. Covariates were age and WML load (at the respective time point)

		Number of WML voxels, mean ± SD [range]	*β*	Adjusted odds ratio [95% CI]	Std. error	*z*-value	*p*-value (uncorr.)
Baseline	Prolonged *T*_1_	691 ± 684^§§§^ [20–2897]	0.822	2.27 [1.07, 4.79]	0.377	2.18	0.029*
	Supramedian *T*_1_	1987 ± 1846^§§§^ [107–7864]	1.160	3.19 [1.05, 9.47]	0.554	2.09	0.036*
One-year follow-up	Prolonged *T*_1_	867 ± 767^§§§^ [40–3166]	0.996	2.71 [1.25, 6.07]	0.393	2.53	0.011*
	Supramedian *T*_1_	2451 ± 2047^§§§^ [163–8883]	1.554	4.73 [1.56, 14.77]	0.567	2.74	0.006**

^*^ and ** indicate significant relationship with EDSS at the *p* < 0.05 and *p* < 0.01 level, respectively

^§§§^Significant change in number of prolonged/supramedian WML voxels over 1 year at *p* < 0.001 level (paired *t*-test)

*β*, standardised beta coefficient; *CI*, confidence interval

**Table 5:** Relationship between prolonged/supramedian *T*_1_ white matter lesion (WML) voxel counts and 1-year change in disability in newly diagnosed relapsing-remitting multiple sclerosis (*n* = 62). Results are shown for binomial logistic regression models investigating the relationship between (a) baseline and (b) 1-year change in prolonged/supramedian *T*_1_ voxel counts and 1-year change in dichotomised Expanded Disability Status Scale (EDSS) score (stable/improving vs worsening EDSS [< / >  = 0.5 point increase]). WML voxels were classed as prolonged *T*_1_ if *T*_1_ ≥ 2.00 s and classed (non-exclusively) as supramedian if *T*_1_ ≥ the mean average of the baseline cohort median WML *T*_1_. Model covariates were ^§^age and lesion load; ^§§^age, baseline voxel count, 1-year change in WML load and disease-modifying treatment status at follow-up (untreated versus treated)

		*β*	Adjusted odds ratios [95% CI]	Std. error	*Z*-value	*p*-value (uncorr.)
Baseline WML voxel count^§^	Prolonged *T*_1_	0.097	1.10 [0.41, 3.11]	0.497	0.20	0.845
	Supramedian *T*_1_	0.055	1.06 [0.26, 4.01]	0.674	0.08	0.935
One-year change in WML voxel count^§§^	Prolonged *T*_1_^a^	1.091	2.98 [1.26, 8.75]	0.482	2.26	0.024*
	Prolonged *T*_1_^b^	0.971	2.64 [1.25, 6.72]	0.42	2.33	0.020*
	Supramedian *T*_1_^a^	1.830	6.23 [1.95, 30.68]	0.687	2.67	0.008**
	Supramedian *T*_1_^b^	1.377	3.96 [1.46, 13.92]	0.566	2.43	0.015*

^a^Including new lesion voxels forming over the 1-year follow-up period

^b^Excluding WML forming over 1 year

Asterisks indicate significant at *p* < 0.05 (*) and *p* < 0.01 (**) levels

*β*, standardised beta coefficient; *CI*, confidence interval; *uncorr.*, uncorrected; *WML*, white matter lesions

## Discussion

Brain *T*_1_, and its relationship with clinical worsening, was quantified longitudinally in a newly diagnosed RRMS cohort. Longitudinal cohort-wide changes in median *T*_1_ were not observed for GM, NAWM or WML. There was significant *T*_1_ heterogeneity in WML; WML voxels with more marked *T*_1_ prolongation increased in number over time, and were associated with disability at baseline and 1-year follow-up. Greater *T*_1_ increases in normal-appearing brain and prolonged *T*_1_ WML components also contributed significantly to evolving disability in the year following RRMS diagnosis.

### *T*_*1*_ variation

Measured* T*_1_ was higher than literature values, [17, 22] which is attributable to differences in acquisition methods. Average WML *T*_1_ values were higher than NAWM, consistent with demyelination [31]. As with previous findings in RRMS, [22] wide variance in WML *T*_1_ and substantial overlap with cGM *T*_1_ values suggest heterogeneous myelin loss; [12] in our cohort, average WML *T*_1_ was lower than cGM, which may reflect the earlier disease stage and less severe WML damage. Medial temporal *T*_1_ was significantly higher than other tissues; measurements in this region are likely confounded by tissue interface effects, and therefore unreliable.

Demyelination appears to be the major determinant of *T*_1_ prolongation in white matter, [31] except within fully demyelinated WML where there is a ‘myelin floor effect’, and axonal content dominates variation in *T*_1_ [12, 32]. Increased tissue water content, [11] due to neuroinflammation or oedema, [33, 34] may also increase *T*_1_. Subtle *T*_*1*_ prolongation in NAWM is therefore likely to represent demyelination, whereas more markedly prolonged *T*_*1*_ components within WML may result from both myelin and axonal loss. Myelin and axonal content are nonetheless strongly associated, [31] and we therefore use the term microstructural damage to reflect the difficulty in establishing their individual contributions to disability in WML and normal-appearing brain.

Our multiple threshold approach in WML is similar to methods applied previously, [22, 35] and analogous to histogram-based analyses [35, 36]. Our findings indicate varying degrees of microstructural damage across individuals at diagnosis, independent of WML load, which accords with lesion variation reported in more advanced disease across MS subtypes, [35, 37–39] and from other microstructure-sensitive methods [6–8].

### Longitudinal change in *T*_1_ and prolonged WML *T*_1_ components

We found no significant longitudinal median *T*_1_ change in grey or white matter regions over the year following diagnosis, in line with previous serial *T*_1_ measurements in early RRMS [5]. A groupwise reduction in WML *T*_1_ over time was attributable to new or enlarging WML; when only WML voxels that were present at baseline were included, no significant *T*_1_ change was found. New WML voxels with less tissue damage developing over the follow-up period therefore appear to drive the cohort-wide WML *T*_1_ decrease, rather than remyelination. WML *T*_1_ reduction over time in early RRMS has been reported previously, [19] and this potential confound should be considered for future study design.

Marked intersubject variability in the proportions of higher WML *T*_1_ components guided our focus on WML voxels with supramedian *T*_1_ and values over 2.00 s; both aim to identify more disrupted microstructure [12]. We found that the number of thresholded *T*_1_ WML voxels increased significantly over 1 year following RRMS diagnosis, indicating increasing microstructural damage within existing WML. To our knowledge, this study is the first to examine the longitudinal evolution of prolonged *T*_1_ components, although thresholding approaches using *T*_1_ [12, 22] and normalised *T*_1_-weighted images [35] have been applied cross-sectionally in MS.

### *T*_1_ and clinical disability

Although positive associations between clinical disability and *T*_1_ within NAWM [14, 21] and WML [14] have been described previously, we did not find an association between cross-sectional median *T*_1_ measures and clinical disability. Previous studies were in mixed MS subtypes at varying disease stages however, and findings varied; [40] the earlier disease stage of our more homogeneous RRMS cohort may account for these differences.

Conversely, cross-sectional prolonged *T*_1_ and supramedian WML voxel counts were associated with EDSS score at the time of diagnosis, a relationship that persisted at 1-year follow-up. The significant increase seen in these WML components over time was also associated with EDSS worsening. This is in line with the literature, where thresholded *T*_1_ has been shown to provide disability-relevant information additional to visual assessment of ‘*T*_1_-w hypointense’ WML in more advanced disease [22]. Our data suggest that prolonged *T*_1_ WML components contribute disproportionately to clinical disability in early RRMS, which may reflect both demyelination and axonal loss [12]. Thresholded WML *T*_1_ measures may therefore provide useful disease stratification in early RRMS.

Longitudinal increases in median *T*_1_ in WML tissue present at baseline in patients with worsening disability, and decreases in those with stable or improving disability, are consistent with disability-relevant microstructural damage and remyelination, respectively. An earlier study did not find any association between a derived WML load-weighted *T*_1_ measure and 1-year change in EDSS score in RRMS, [10] which may reflect differences in cohort size, disease duration and analytical approach.

In our cohort, greater 1-year increase in median *T*_1_ in NAWM, cGM and thalamus was also associated with EDSS worsening, whereas *T*_1_ was relatively stable or decreasing in participants with stable or improving EDSS, resulting in an absence of net cohort-wide *T*_1_ change in GM or NAWM. *T*_1_ therefore captures disability-relevant demyelination that is not visible on conventional MRI, and the direction of *T*_1_ change over 1 year may indicate disability trajectory in early MS.

None of our baseline measures were predictive of EDSS worsening over the following year. High individual variance in baseline brain microstructural integrity, despite a relatively homogeneous diagnosis point, and limited 1-year clinical worsening following RRMS diagnosis may be limiting factors.

### Limitations

*T*_1_ quantification may be useful as a group-level patient stratifier for targeted clinical trials; the small magnitude of changes and reproducibility of measures may, however, limit application in individual patients. VFA acquisition for *T*_1_ approximation is rapid and readily implemented, but does not correct for B_1_ inhomogeneities, which may reduce sensitivity to biological change and introduce spatial bias, although unlikely to substantially effect longitudinal measures. WML were segmented on 3-mm 2D FLAIR slices rather than 3D FLAIR due to better contrast resolution, which may introduce partial volume effects. Although all participants were recruited within 6 months of diagnosis, the relationship with true disease onset is variable and difficult to determine reliably; [41] diagnosis was therefore chosen as a pragmatic and measurable time point. Effects of intercurrent treatment are likely to be minimal: MRI was performed a minimum of 3 weeks post-steroid treatment; DMT status was not related to disability outcome; however, treatment variation in this cohort precludes meaningful sensitivity analysis. Innate EDSS limitations are well-recognised [42]. Moreover, 1-year change in EDSS score showed significant intersubject variability (Fig. S3); overall disability worsening was minor, and confounded by improvement following the clinical episode that initiated RRMS diagnosis. Five-year follow-up of the FutureMS cohort [23] will allow more reliable evaluation of *T*_1_ measures as predictors of disease trajectory.

## Conclusion

Quantitative *T*_1_ mapping is sensitive to disability-relevant microstructural changes in the year following RRMS diagnosis. *T*_1_ thresholding approaches demonstrate heterogeneity of microstructural damage in WML; WML components with prolonged *T*_1_ increase with time, and are significantly associated with clinical disability. Widespread subtle demyelination can also be detected in NAWM and GM in patients with worsening disability. Further studies in well-characterised RRMS cohorts over longer time periods will help clarify the utility of *T*_1_ mapping as a disease stratifier, and for potential application in future therapeutic trials.

### Supplementary Information

Below is the link to the electronic supplementary material.

Supplementary file1 (PDF 661 KB)

## Data Availability

Researchers may request access to anonymised patient data from FutureMS-1 following our standard procedures. To submit an access, request please contact future-ms@ed.ac.uk. Proposals will be reviewed and approved by the FutureMS steering committee. A signed data sharing agreement will then be issued. Data will be available as soon as possible after the first publication of the results.

## Abbreviations

cGM: Cortical grey matter
DGM: Deep grey matter
DMTs: Disease-modifying therapies
EDSS: Expanded Disability Status Scale
FDR: False discovery rate
FLAIR: Fluid-attenuated inversion recovery
GM: Grey matter
MRI: Magnetic resonance imaging
NAWM: Normal-appearing white matter
RRMS: Relapsing-remitting multiple sclerosis
*T*_1_: Spin-lattice relaxation time
VFA: Variable flip angle
WML: White matter lesions

## Declarations

### Guarantor

The scientific guarantor of this publication is Professor Adam Waldman.

### Statistics and biometry

One of the authors has significant statistical expertise.

### Study subjects or cohorts overlap

The current article focuses on quantitative *T*_*1*_ data from a sub-cohort of FutureMS participants who received extended microstructural MRI, and test-retest healthy control *T*_*1*_ data, which have not been reported on previously.

FutureMS studies which have been previously published:

1. Glasmacher SA, Kearns PKA, Hassan Z, et al (2022) The influence of disease-modifying therapy on hidden disability burden in people with newly diagnosed relapsing-remitting multiple sclerosis. Multiple Sclerosis and Related Disorders. https://doi.org/10.1016/j.msard.2022.103837.

2. Chang Y-T, Kearns PKA, Carson A, et al (2023) Network analysis characterizes key associations between subjective fatigue and specific depressive symptoms in early relapsing-remitting multiple sclerosis. Multiple Sclerosis and Related Disorders. https://doi.org/10.1016/j.msard.2022.104429.

3. Kearns PKA, Martin SJ, Chang J, et al (2022) FutureMS cohort profile: a Scottish multicentre inception cohort study of relapsing-remitting multiple sclerosis. BMJ Open. https://doi.org/10.1136/bmjopen-2021-058506.

4. York EN, Meijboom R, Thrippleton MJ, et al (2022) Longitudinal microstructural MRI markers of demyelination and neurodegeneration in early relapsing-remitting multiple sclerosis: magnetisation transfer, water diffusion and g-ratio. Neuroimage: Clinical. https://doi.org/10.1016/j.nicl.2022.103228.

5. York EN, Martin SJ, Meijboom R, et al (2021) MRI-derived g-ratio and lesion severity in newly diagnosed multiple sclerosis. Brain Commun. https://doi.org/10.1093/braincomms/fcab249.

6. Ng Kee Kwong KC, Mollison D, Meijboom R, et al (2022) Rim lesions are demonstrated in early relapsing-remitting multiple sclerosis using 3 T-based susceptibility-weighted imaging in a multi-institutional setting. Neuroradiology. https://doi.org/10.1007/s00234-021-02768-x.

7. Chen Y, Larraz J, Wong M, et al (2022) Longitudinal retinal imaging study of newly diagnosed relapsing-remitting multiple sclerosis in Scottish population: baseline and 12 months follow-up profile of FutureMS retinal imaging cohort. BMJ Open Ophthalmology. https://doi.org/10.1136/bmjophth-2022-001024.

8. Meijboom R, York EN, Kampaite A, et al Patterns of brain atrophy in recently-diagnosed relapsing-remitting multiple sclerosis. PLoS One. 2023;18(7):e0288967. Published 2023 Jul 28. https://doi.org/10.1371/journal.pone.0288967

### Methodology

- • prospective
- • observational
- • performed at one institution
