# Supplementary material for: Quantitative T1 brain mapping in early relapsing-remitting multiple sclerosis: longitudinal changes, lesion heterogeneity and disability
Source: Eur Radiol. 2023 Nov 9;34(6):3826–39. doi: 10.1007/s00330-023-10351-6 (PMC11166797; doi:10.1007/s00330-023-10351-6)
Supplement: Supplementary file 1 — Supplementary file1 (PDF 661 KB) [file 330_2023_10351_MOESM1_ESM.pdf]

Quantitative T1 brain mapping in early relapsing-remitting multiple sclerosis: longitudinal changes, lesion heterogeneity and disability

Electronic Supplementary Material

Supplementary Tables

Table S1: part of the imaging protocol used in the FutureMS multi-centre study. FutureMS parameters described in full in Meijboom et al. [1].

Sequence	Multi-echo spoiled gradient echo	T_1-weighted (MPRAGE)	2D FLAIR (PROPELLER)
**Mode**	3D	3D	2D
**Field of View (mm)**	224 (SI) x 241 (AP)	256	250
**Orientation**	Sagittal	Sagittal	Axial
**Repetition Time (ms)**	30	2500	9500
**Echo Time (ms)**	1.54/4.55/8.49	2.26	120
**Inversion Time (ms)**	-	1100	2400
**Flip Angle (degrees)**	5	7	150
**Gap (mm)**	0	-	0
**Matrix (mm)**	160 x 172	256 x 256	256 x 256
**Voxel Size (mm)**	1.4 x 1.4 x 1.4	1 x 1 x 1	1 x 1 x 3
**Slices**	128	176	60
**Acceleration Factor (in plane x slice)**	2 x 1	2 x 1	2 x 1
**Acquisition Time (m:ss)**	6:14/6:14	5:59	4:47

Table S2: Overview of regression models to determine the relationship between tissue microstructure and disability in recently diagnosed relapsing-remitting multiple sclerosis. Continuous variables were centred and scaled for regression analyses.

		Measure of interest			
**Aim**	**Analysis**	**Measure**	**Tissue**	**Covariates**	**Outcome**
Cross-sectional relationship between tissue microstructure and disability at point of diagnosis	Ordinal logistic regression	Baseline median T1	NAWM, cGM, WML, medial temporal regions, basal ganglia, thalami, global DGM	Age; baseline lesion load	Baseline EDSS score
		Baseline number of prolonged T1 voxels	WML		
		Baseline number of supramedian T1 voxels	WML		
Relationship between baseline tissue microstructure and longitudinal change in disability	Binomial logistic regression	Baseline median T1	NAWM, cGM, WML, medial temporal regions, basal ganglia, thalami, global DGM	Age; baseline lesion load	One-year change in EDSS (dichotomised as either worsening EDSS, defined as ≥ 0.5 points, or stable/improving)
		Number of prolonged T1 voxels	WML		
		Number of supramedian T1 voxels	WML		
Relationship between longitudinal change in tissue microstructural and longitudinal change in disability	Binomial logistic regression	One-year change in median T1	NAWM, cGM, WML, medial temporal regions, basal ganglia, thalami, global DGM; WML voxels present at baseline	Age; baseline median T1; change in lesion load; DMT status (defined as untreated or treated); interaction terms when significant (α = 0.05)	One-year change in EDSS (dichotomised as either worsening EDSS, defined as ≥ 0.5 points, or stable/improving)
			WML; WML voxels present at baseline		
			WML; WML voxels present at baseline		
Cross-sectional relationship between WML tissue heterogeneity and disability at one year follow-up	Ordinal logistic regression	Number of prolonged T1 voxels at one year follow-up	WML	Age; lesion load at follow-up	EDSS score at one-year follow-up
		Number of supramedian T1 voxels at one-year follow-up	WML		

DMT: disease-modifying therapy; EDSS: Expanded Disability Status Score; global DGM: global deep grey matter; NAWM: normal-appearing white matter; cGM: cortical grey matter; WML: white matter lesions.

Table S3: treatment with disease-modifying therapies (DMTs) at one-year follow-up. All participants were untreated with DMTs at baseline.

DMTs	All participants<br>n = 62	Stable/improving<br>n = 25	Worsening<br>n = 37
Alemtuzumab	2	1	1
Beta interferon	3	1	2
Dimethyl fumarate	23	11	12
Dimethyl fumarate; azathioprine	1	1	0
Fingolimod	1	0	1
Glatiramer acetate	5	2	3
Natalizumab	1	1	0
Other	2	0	2
Never	24	8	16

Table S4: Brain tissue median T_1 test-retest results for healthy control participants (n=11). 95 % of median T_1 measurements in healthy participants would be expected to fall within Bland-Altman limits of agreements. S-values are given for one-sample sign tests.

Brain Tissue	T_1 (ms), mean ± SD			Mean diff. (ms) ± Bland-Altman limits of agreement	Median diff. (ms) [95% CI]	s-value	p-value (uncorr.)
	Time point 1	Time point 2	Mean				
**White Matter**	1102 ± 69	1074 ± 49	1088 ± 51	-28 ± 120	-44 [-90, 41]	5	1
**Cortical Grey Matter**	1632 ± 83	1610 ± 67	1621 ± 67	-22 ± 133	-28 [-98, 43]	5	1
**Global Deep Grey Matter**	1813 ± 102	1782 ± 67	1798 ± 73	-31 ± 180	-37 [-125, 62]	5	1
**Basal Ganglia**	1694 ± 101	1662 ± 61	1678 ± 68	-32 ± 187	-24 [-128, 64]	5	1
**Medial Temporal Region**	2228 ± 107	2203 ± 74	2216 ± 81	-25 ± 171	-11 [-101, 50]	4	0.549
**Thalami**	1817 ± 114	1789 ± 83	1803 ± 88	-28 ± 184	-46 [-121, 53]	5	1

CI: confidence interval; SD: standard deviation; diff.: difference (time point 2 – time point 1); uncorr.: uncorrected for multiple comparisons.

Table S5: Results of cross-sectional ordinal logistic regression models investigating the relationship between median baseline T_1 in each tissue and baseline Expanded Disability Status Scale (EDSS) scores.

Brain Tissue	β	Adjusted Odds Ratio [95% CI]	Std. Error	Z-value	p-value (uncorr.)	p-value (FDR-adj.)
**White Matter Lesions**	0.613	1.85 [1.15, 3.03]	0.245	2.50	**0.012**	0.084
**Normal-appearing White Matter**	0.079	1.08 [0.67, 1.77]	0.248	0.32	0.751	0.922
**Cortical Grey Matter**	0.184	1.20 [0.73, 2.00]	0.255	0.72	0.470	0.922
**Global Deep Grey Matter**	0.063	1.06 [0.66, 1.74]	0.249	0.25	0.801	0.922
**Basal Ganglia**	0.080	1.08 [0.66, 1.79]	0.253	0.31	0.753	0.922
**Medial Temporal Region**	-0.176	0.84 [0.53, 1.33]	0.232	-0.76	0.448	0.922
**Thalami**	-0.024	0.98 [0.61, 1.58]	0.243	-0.10	0.922	0.922

Age and lesion load were included as covariates. Adjusted odds ratios are for standardized data; β: standardized beta coefficient; CI: confidence interval; FDR: False Detection Rate.

Table S6: Results of the binomial logistic regression models investigating the relationship between the median baseline T_1 measures in each tissue and one-year change in Expanded Disability Status Scale (EDSS) score (stable/improving vs worsening EDSS >0.5 points over one year).

Brain Tissue	β	Adjusted Odds Ratio [95% CI]	Std. Error	Z-value	p-value (uncorr.)	p-value (FDR-adj.)
White Matter Lesions	0.310	1.36 [0.79, 2.45]	0.284	1.09	0.276	0.859
Normal-appearing White Matter	0.275	1.32 [0.75, 2.39]	0.291	0.946	0.344	0.859
Cortical Grey Matter	0.110	1.12 [0.63, 1.98]	0.286	0.39	0.700	0.957
Global Deep Grey Matter	0.082	1.09 [0.61, 1.92]	0.289	0.28	0.777	0.957
Basal Ganglia	0.259	1.30 [0.73, 2.32]	0.288	0.90	0.368	0.859
Medial Temporal Region	0.059	0.94 [0.51, 1.66]	0.288	-0.21	0.837	0.957
Thalami	0.016	1.02 [0.56, 1.82]	0.294	0.05	0.957	0.957

Covariates included were age and lesion load. Adjusted odds ratios are for standardized data; β: standardized beta coefficient; CI: confidence interval.

Table S7: Brain tissue median T_1 summary statistics, grouped by dichotomised Expanded Disability Status Scale (EDSS) change over one year (worsening EDSS: ≥0.5 points).

Brain Tissue	T_1 (ms), mean ± SD				Mean difference (ms) [95% CI]	
	stable/improving EDSS		worsening EDSS		stable/ improving EDSS	worsening EDSS
	baseline	follow-up	baseline	follow-up		
**White Matter Lesions[a]**	1442±160	1390±122	1476±175	1458±157	-52 [-90, -13]	-18 [-42, 7]
**White Matter Lesions[b]**	1442±160	1415±154	1476±175	1486±170	-27 [-64, 10]	10 [-14, 33]
**Normal-appearing White Matter**	1065±66	1060±53	1081±59	1103±62	-5 [-27, 17]	22 [3,42]
**Cortical Grey Matter**	1608±76	1596±61	1617±79	1643±66	-13 [-43, 18]	26 [3, 49]
**Global Deep Grey Matter**	1790±86	1773±92	1802±99	1826±79	-17 [-54, 20]	24 [-7, 55]
**Basal Ganglia**	1666±85	1647±88	1688±100	1706±85	-19 [-54, 17]	18 [-12, 48]
**Medial Temporal Region**	2214±75	2200±105	2220±128	2236±88	-14 [-54, 26]	16 [-19, 51]
**Thalami**	1779±85	1765±85	1786±94	1820±84	-14 [-49, 21]	34 [1, 68]

White matter lesions: [a] includes any voxels reclassified as lesional at one-year follow-up; [b]only includes lesions present at baseline. CI: confidence interval; SD: standard deviation.

Supplementary Figures

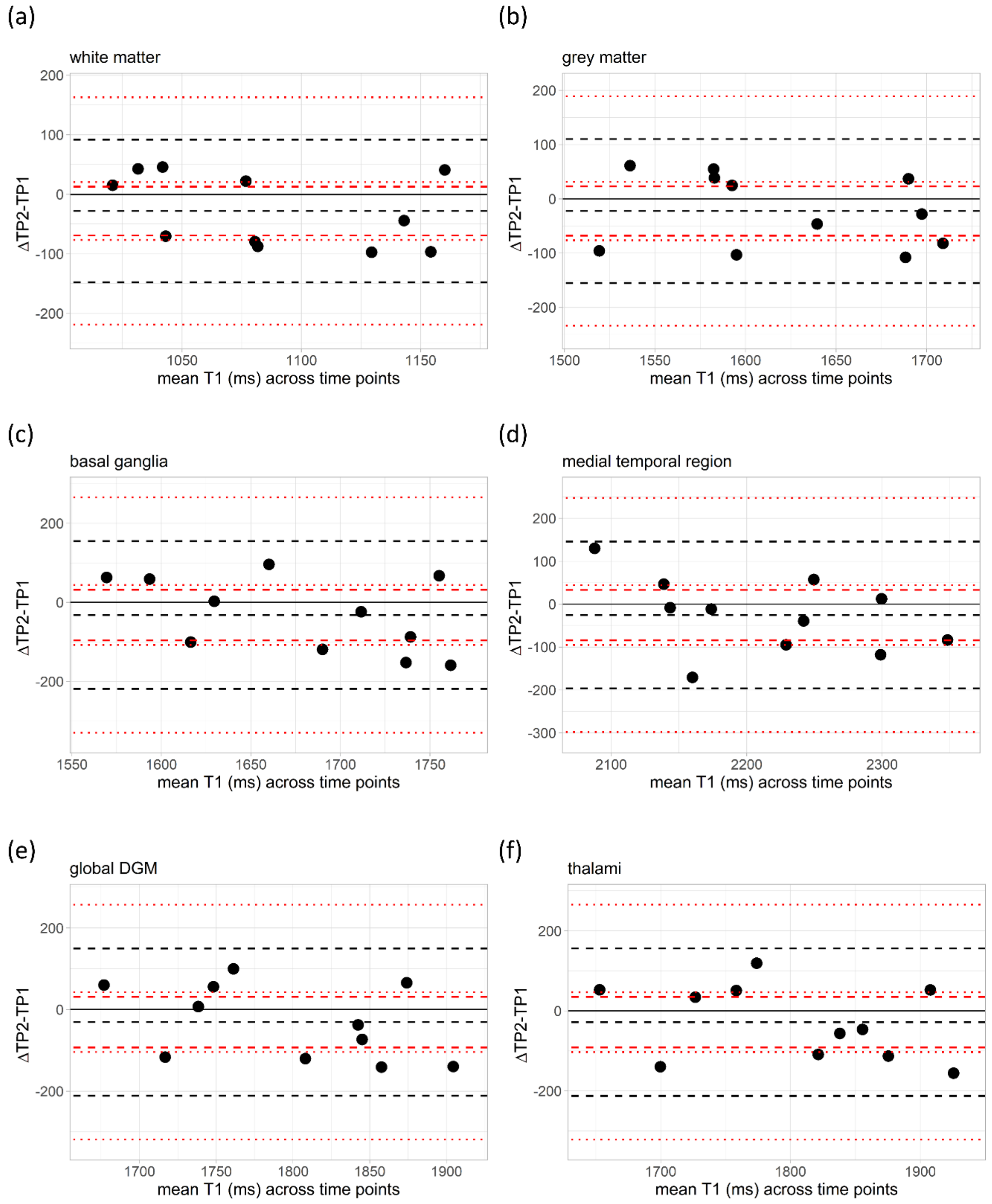

Figure S1: test-retest reliability was established by calculating Bland-Altman limits of agreement for T_1 in healthy control brain regions (BlandAltmanLeh R package) to compare with longitudinal change in patient T_1. Black dashed lines: mean difference and limits of agreement in which 95% of measurements in healthy subjects would be expected to fall; red dotted lines: 95% confidence intervals for limits of agreement; red dashed lines: 95% confidence interval for mean difference (see **Table S3** for numeric data). DGM: deep grey matter; TP1/2: time point 1 and 2; Δ difference in median T_1 (ms) between time points.

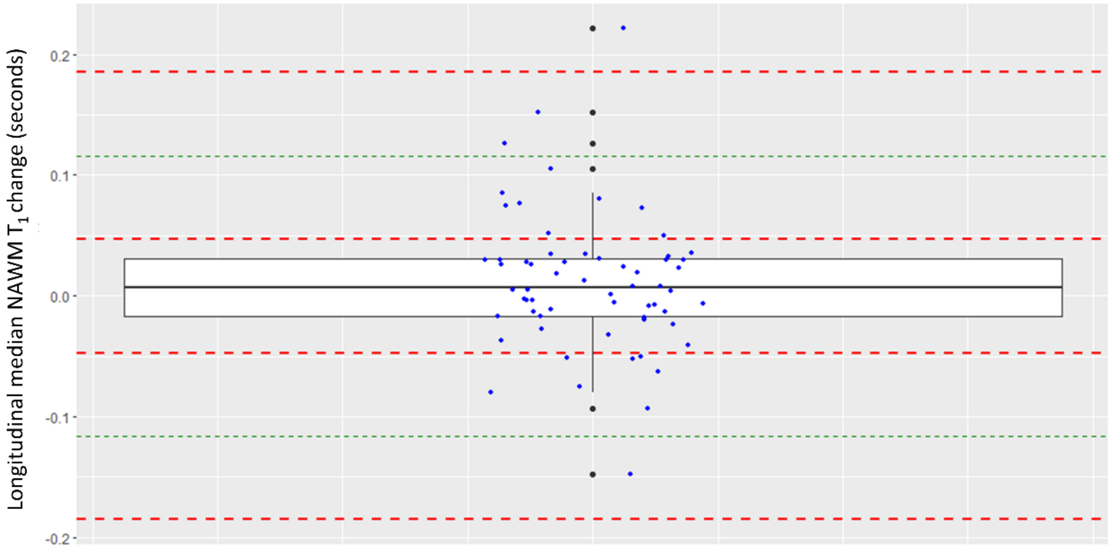

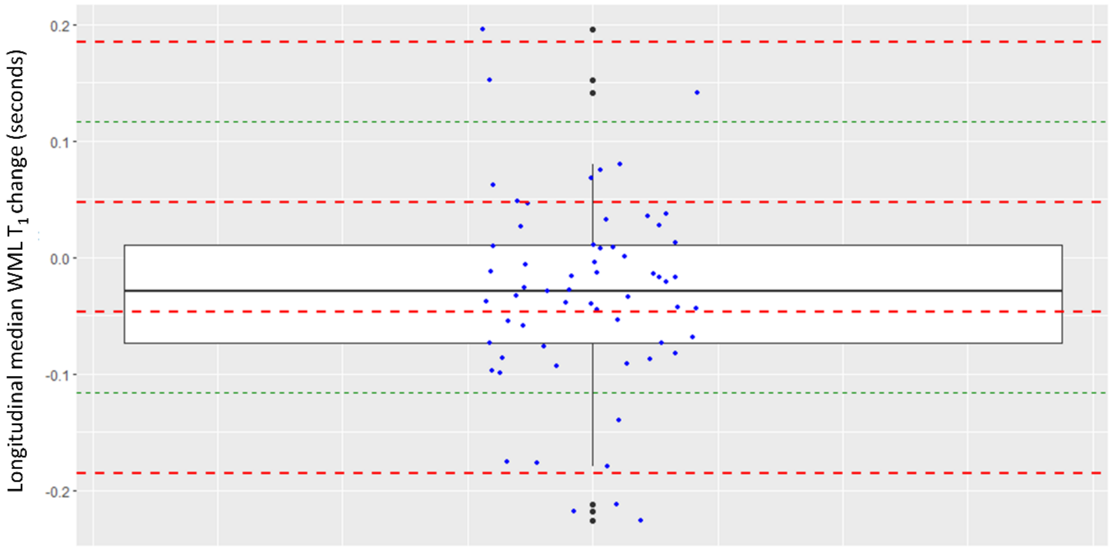

Figure S2: One-year changes in median normal-appearing white matter T_1 (top) and median white matter lesion T_1 in relapsing-remitting multiple sclerosis (RRMS) group. The Bland-Altman limits of agreement, as calculated on healthy control white matter data, are superimposed on both plots using a green dashed line. The confidence intervals have been similarly superimposed using a red dashed line. Each blue dot represents one RRMS participant in the study cohort.

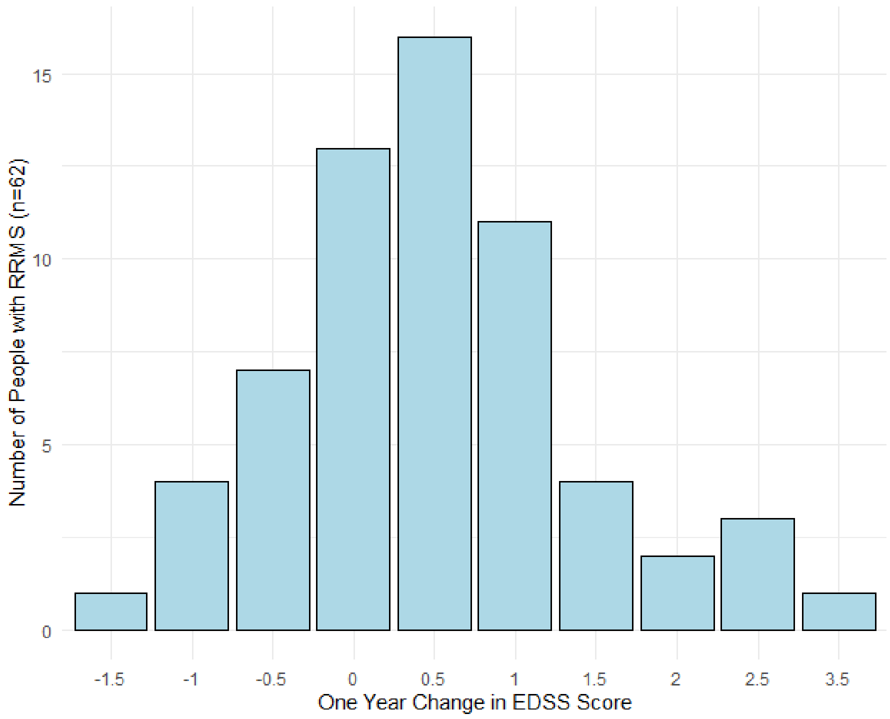

Figure S3: histogram showing the one-year change in Expanded Disability Status Scale (EDSS) scores of this study's cohort. Despite a median longitudinal increase of +0.5, many participants show clinical improvement (as shown by a decrease in EDSS score) and significant variability in one-year change in EDSS score was observed across our early relapsing-remitting multiple sclerosis cohort.

References

1. Meijboom R, Wiseman SJ, York EN et al (2022) Rationale and design of the brain magnetic resonance imaging protocol for FutureMS: a longitudinal multi-centre study of newly diagnosed patients with relapsing-remitting multiple sclerosis in Scotland [version 1; peer review: 2 approved] Wellcome Open Research, 7:94, https://doi.org/10.12688/wellcomeopenres.17731.1

STROBE Statement—Checklist of items that should be included in reports of ***cohort studies***

	Item No	Recommendation	Page No
**Title and abstract**	1	(*a*) Indicate the study's design with a commonly used term in the title or the abstract	1
		(*b*) Provide in the abstract an informative and balanced summary of what was done and what was found	1-2
**Introduction**			
Background/rationale	2	Explain the scientific background and rationale for the investigation being reported	4-5
Objectives	3	State specific objectives, including any prespecified hypotheses	5
**Methods**			
Study design	4	Present key elements of study design early in the paper	6
Setting	5	Describe the setting, locations, and relevant dates, including periods of recruitment, exposure, follow-up, and data collection	6
Participants	6	(*a*) Give the eligibility criteria, and the sources and methods of selection of participants. Describe methods of follow-up	6
		(*b*) For matched studies, give matching criteria and number of exposed and unexposed	
Variables	7	Clearly define all outcomes, exposures, predictors, potential confounders, and effect modifiers. Give diagnostic criteria, if applicable	6-9
Data sources/ measurement	8*	For each variable of interest, give sources of data and details of methods of assessment (measurement). Describe comparability of assessment methods if there is more than one group	6-9
Bias	9	Describe any efforts to address potential sources of bias	8-9
Study size	10	Explain how the study size was arrived at	6
Quantitative variables	11	Explain how quantitative variables were handled in the analyses. If applicable, describe which groupings were chosen and why	6-8, Table S2
Statistical methods	12	(*a*) Describe all statistical methods, including those used to control for confounding	7-9, Table S2
		(*b*) Describe any methods used to examine subgroups and interactions	8-9, Table S2
		(*c*) Explain how missing data were addressed	6
		(*d*) If applicable, explain how loss to follow-up was addressed	6
		(*e*) Describe any sensitivity analyses	16

**Results**			
Participants	13*	(a) Report numbers of individuals at each stage of study—eg numbers potentially eligible, examined for eligibility, confirmed eligible, included in the study, completing follow-up, and analysed	Fig 1
		(b) Give reasons for non-participation at each stage	Fig 1
		(c) Consider use of a flow diagram	Fig 1
Descriptive data	14*	(a) Give characteristics of study participants (eg demographic, clinical, social) and information on exposures and potential confounders	10, Table 1, Table S3
		(b) Indicate number of participants with missing data for each variable of interest	NA – all complete
		(c) Summarise follow-up time (eg, average and total amount)	Table 1
Outcome data	15*	Report numbers of outcome events or summary measures over time	Tables 1, 2 and 4; Table S7; p10
Main results	16	(*a*) Give unadjusted estimates and, if applicable, confounder-adjusted estimates and their precision (eg, 95% confidence interval). Make clear which confounders were adjusted for and why they were included	Table 3-5; Tables S4-S6
		(*b*) Report category boundaries when continuous variables were categorized	8
		(*c*) If relevant, consider translating estimates of relative risk into absolute risk for a meaningful time period	NA
Other analyses	17	Report other analyses done—eg analyses of subgroups and interactions, and sensitivity analyses	10-12
**Discussion**			
Key results	18	Summarise key results with reference to study objectives	13
Limitations	19	Discuss limitations of the study, taking into account sources of potential bias or imprecision. Discuss both direction and magnitude of any potential bias	15-16
Interpretation	20	Give a cautious overall interpretation of results considering objectives, limitations, multiplicity of analyses, results from similar studies, and other relevant evidence	13-16
Generalisability	21	Discuss the generalisability (external validity) of the study results	16-17
**Other information**			
Funding	22	Give the source of funding and the role of the funders for the present study and, if applicable, for the original study on which the present article is based	17-18

*Give information separately for exposed and unexposed groups.

Note: An Explanation and Elaboration article discusses each checklist item and gives methodological background and published examples of transparent reporting. The STROBE checklist is best used in conjunction with this article (freely available on the Web sites of PLoS Medicine at http://www.plosmedicine.org/, Annals of Internal Medicine at http://www.annals.org/, and Epidemiology at http://www.epidem.com/). Information on the STROBE Initiative is available at http://www.strobe-statement.org.
